# Molecular architecture of the Jumonji C family histone demethylase KDM5B

**DOI:** 10.1038/s41598-019-40573-y

**Published:** 2019-03-11

**Authors:** Jerzy Dorosz, Line Hyltoft Kristensen, Nanda G. Aduri, Osman Mirza, Rikke Lousen, Saskia Bucciarelli, Ved Mehta, Selene Sellés-Baiget, Sara Marie Øie Solbak, Anders Bach, Pablo Mesa, Pablo Alcon Hernandez, Guillermo Montoya, Tam T. T. N. Nguyen, Kasper D. Rand, Thomas Boesen, Michael Gajhede

**Affiliations:** 1 Biostructural Research, Department of Drug Design and Pharmacology, Faculty of Health and Medical Sciences, University of Copenhagen, Jagtvej 162, 2100 Copenhagen, Denmark; 2 Medicinal Chemistry Research, Department of Drug Design and Pharmacology, Faculty of Health and Medical Sciences, University of Copenhagen, Jagtvej 162, 2100 Copenhagen, Denmark; 3 Protein Structure & Function Programme, Macromolecular Crystallography Group, Novo Nordisk Foundation Center for Protein Research, Faculty of Health and Medical Sciences, University of Copenhagen, Blegdamsvej 3B, Copenhagen, 2200 Denmark; 4 Department of Pharmacy, Faculty of Health and Medical Sciences, University of Copenhagen, Universitetsparken 2, 2100 Copenhagen, Denmark; 5 Interdisciplinary Nanoscience Center, Aarhus University, Gustav Wieds Vej 14, 8000 Aarhus, Denmark

## Abstract

The full length human histone 3 lysine 4 demethylase KDM5B (PLU-1/Jarid1B) has been studied using Hydrogen/Deuterium exchange mass spectrometry, homology modelling, sequence analysis, small angle X-ray scattering and electron microscopy. This first structure on an intact multi-domain Jumonji histone demethylase reveal that the so-called PLU region, in the central region of KDM5B, has a curved α-helical three-dimensional structure, that acts as a rigid linker between the catalytic core and a region comprising four α-helices, a loop comprising the PHD2 domain, two large intrinsically disordered loops and the PHD3 domain in close proximity. The dumbbell shaped and curved KDM5B architecture observed by electron microscopy is complementary to the nucleosome surface and has a striking overall similarity to that of the functionally related KDM1A/CoREST complex. This could suggest that there are similarities between the demethylation mechanisms employed by the two histone 3 lysine 4 demethylases at the molecular level.

## Introduction

In gene regulation accessibility to promotor regions for the transcriptional machinery is now known be of major importance^1^. Posttranslational modifications (PTMs) of the unstructured N-terminals of histones are important regulators of the accessibility, where important modifications are methylations and acetylations of lysine residues^2^. Enzymes are classified as readers, writers or erasers when they read, introduce or remove accessibility controlling PTMs, respectively.

The histone lysine demethylase (HDM) family is a large group of erasers that comprises a total of around 30 enzymes^3^. Dependent on their specificity towards the histone tail they can be divided in subfamilies^4^. HDM family enzymes are known to be controllers of development and cell fate decisions^4^. With these functions, they are also involved in the development of cancer^5^.

The FAD dependent KDM1A (LSD1) and KDM1B (LSD2) are histone 3 lysine 4 di- and mono- (H3K4me2/1) demethylases that are incapable of demethylating H3K4me3. KDM1A is the most studied and predominately found in nanomolar affinity complexes with REST co-repressor proteins^6^. The crystal structure of KDM1A revealed an elongated structure including the flavin binding catalytic domain and a helical so-called tower domain^7^. The structure of a KDM1A/CoREST complex^8^ further showed that CoREST forms a triple helix coiled coil structure with the KDM1A tower domain. A DNA binding SANT domain (SANT2) is hereby positioned away from the catalytic domain at the other end of the tower domain. With this architecture the DNA binding ability of the complex is separated from the catalytic functionality by a long linker. Later studies have led to a model for the molecular mechanism of KDM1A/CoREST mediated nucleosome demethylation^9^. Here demethylation is initiated by relative low affinity SANT2 mediated unspecific DNA binding, a process that also detaches histone tails from the nucleosome. The complex brings its catalytic site in correct position for demethylation of the opened H3K4me2/me1 sites by scanning all of the nucleosomal DNA binding sites.

**Figure 1:**
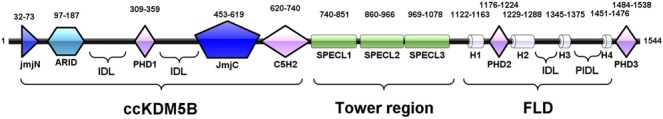
Schematic representation of the domain structure of KDM5B. Domains that have previously been identified are shown with bold edges. Sequence borders are indicated above each domain.

Except for KDM1A and KDM1B, all of the HDM demethylases belong to the family of Jumonji C (JmjC) domain containing iron and α-ketoglutarate (2OG) dependent oxygenases^10^. The KDM5 subfamily comprises four enzymes that all contain a characteristic central PLU region and are capable of demethylating H3K4me3/me2^11,12^. Functionally the 1544 residue KDM5B (PLU-1, Jarid1B) enzyme has been shown to target genes that regulate development and to be involved in neural differentiation^13^. KDM5B contains 7 annotated domains, a JmjN domain, the catalytic JmjC domain, a DNA binding ARID domain, a C_5_HC_2_ zinc finger and three plant homeo domains (PHD1-3)^10^. The JmjN, JmjC, PHD1, ARID and the helical C_5_HC_2_ motif containing (C_5_HC_2_) domains constitute a catalytic core (ccKDM5B) with *in vivo* catalytic activity^14^, see Fig. 1. The specificities of most of the binding domains have been mapped. A KDM5 ARID domain has been shown to recognize specific DNA sequences, and the binding consensus has been mapped to CCGCCC for KDM5A^15^ and GCACA/C for KDM5B^16^. The PHD1 domain binds H3K4me0 strongest and H3K4me1 with only 5-fold lower affinity. The PHD3 has preference for H3K4me3 but also binds the other H3K4 methylation states^17,18^.

The structure of the ccKDM5B with an internal deletion (residues 26–772 with residues 102–373 deleted) has been determined recently and deposited with PDBID 5A1F^19^. The deletion comprises the ARID and the PHD1 domains. Information on the orientation of the ARID domain is, however, available from the highly similar ccKDM5A structure^20^. To date nothing is known about the structure of the C-terminal region of KDM5B.

In order to change the actively transcribed gene H3K4me3 mark to the repressed gene H3K4me0 mark, both the actions of the H3K4me3/me2 KDM5 family enzymes and the H3K4me2/me1 KDM1 family enzymes are required. KDM1A and KDM5B have previously been reported to interact. It was shown that KDM5B interacts physically with the repressive KDM1A:NuRD complex in HeLa and MCF-7 cells, with direct interactions to HDAC1, MBD3 and KDM1A and that H3K4 demethylation by the two enzymes is conducted in a sequential manner^21^. The same study demonstrated that the C-terminal region of KDM5B binds to nucleosomes. The details of these concerted reactions on nucleosomes are, however, still far from clear.

In this study we have investigated the structure and function of KDM5B using a range of different techniques including hydrogen deuterium exchange mass spectrometry (HDX-MS), sequence analysis, molecular modeling, surface plasmon resonance (SPR), small angle X-ray scattering (SAXS) and negative stain electron microscopy (EM). We find that KDM5B, although being a larger molecule, has an architecture that is very similar to that of the KDM1A/CoREST complex. Investigating the *in vitro* nucleosome binding to KDM5B by SPR, suggest that the intact KDM5B binds tightly to nucleosomes.

## Methods

### Protein expression and purification

ccKDM5B residues 1–820 was expressed and purified as previously described^22^. For the full length KDM5B PCR amplified DNA coding for human KDM5B residue 1–1544 was cloned into the pOPINF vector^23^, encoding a N-terminal His6-purification tag that is cleavable by the human rhinovirus 3C protease. Recombinant baculovirus was produced in Sf9 insect cells as previously described^24^ by co-transfecting the KDM5B encoding vector and linearized AcNPV-derived DNA (BD biosciences). Suspension cultures of Hi5 cells in BD Baculogold Max-XP Insect Cell Medium (BD Biosciences) supplemented with 3.5% fetal bovine serum was used in the virus transfections. At a cell density of 1.2–2.4 million/mL infection was done with a MOI of 5–10. The cells were harvested after 40–42 hours. A lysis/equilibration/wash (LEW) buffer comprising 50 mM HEPES pH 7.7, 300 mM NaCl, 5–10% glycerol, 1.5 mM MgCl_2_, 2 mM PMSF 5 mM imidazole and 1 Complete Protease Inhibitor Cocktail tablet (Roche) per 50 mL was used for resuspension. Next, cells were lysed using sonication and centrifuged at 87,000 g for a period of 60 min. The supernatant was filtered and mixed with Talon metal affinity resin (Clontech) suspended in LEW buffer and gently rotated for 2 hours at 6 °C. The resin was washed thoroughly with LEW buffer followed by on column cleavage of the His6 tagged KDM5B with the His-GST-tagged 3C protease. The eluted KDM5B (with the sequence GP added from the cleavage, yielding a protein with a theoretical molecular weight (MW) of 175.8 kDa when assuming no post-translational modifications) was concentrated using ultrafiltration filters (Amicon) to prepare for size exclusion chromatography (SEC). SEC was performed using a HiLoad 26/600 Superdex 200 pg column (GE Healthcare) that had been equilibrated with the running buffer composed of 50 mM HEPES pH 7.7, 300 mM NaCl, 5% glycerol and 1 mM DTT. Expression levels were highly dependent on virus age, cell generation and aeration and typically resulted in 2–4 mg of purified protein/L suspension infected cells. The protein was stored at −80 °C until use. The analytical SEC investigations of the KDM5B concentration elution time dependence was performed using a Superdex 200 5/150 Increase (GE Healthcare) and the same buffer system. The extended tails, relative to that of the SEC run in Figure S1A, observed in the analytical runs suggest that the amount of the 90 kDa species seen in fractions B4 and B5 in Figure S1B increases over time and extended handling.

Nucleosome core particles (NCPs) were produced as previously described^25,26^. In short, recombinant His6 tagged core histones from *Xenopus laevis* were expressed in *E. coli* yielding inclusion bodies. After purification of the inclusion bodies of the single proteins, histone dimer (H2A-H2B) and tetramer (2xH3-H4) were refolded and further assembled into histone octamer. Mono-nucleosomes were then produced by reconstitution of the histone octamer with the’ Widom 601’ DNA sequence^27^ using the salt-gradient dialysis method. The final reconstituted NCPs were further purified by preparative electrophoresis using the PrepCell model 491 (BIO-RAD) equipped with a 19-mm i.d. column and a 8-cm tall 6% polyacrylamide (ratio acrylamide:bis-acrylamide 19:1), 0.2X Tris-borate-EDTA (TBE) buffer gel using 0.2X TBE as running buffer and 20 mM Tris HCl pH 7.5, 1 mM EDTA, 1 mM DTT as elution buffer.

### Analytical size exclusion chromatography (SEC)

KDM5B elution volumes were studied using a Superdex 200 5/150 Increase column (GE Health Care) using an HPLC system (Agilent 1100). KDM5B in concentrations in the range 0.05–2 mg/ml were applied using a buffer of 50 mM HEPES 300 mM NaCl pH 7.7 and 1 mM DTT and a flow rate of 0.25 mL/min. Samples were kept at 5 °C until the application to the column. The column was calibrated using standards from the LMW and HMW kits (Sigma A6103).

### SAXS

SAXS experiments were performed at 7 °C using a BioXolver L (Xenocs) with a GeniX3D X-ray source (wavelength of created X-rays: λ = 1.54 Å) and a motorized detector, allowing to change the sample-detector distance d to cover a broader range of q-values where q = 4π/λ sin(θ/2) is the length of the scattering vector and θ is the scattering angle. The measurements were performed with two different sample-detector distances: d = 571 mm (q = 0.01 Å^−1^–0.5 Å^−1^) and d = 1382 mm (q = 0.005 Å^−1^–0.2 Å^−1^). 7 µl of purified KDM5B at 4 different concentrations (0.75, 1, 1.5 and 1.8 mg/mL) and the corresponding buffer were automatically loaded using the sample handling robot of the BioXolver. Multiple frames of 60 or 120 s were collected (see Table S1 for details), corrected for background radiation, direct beam intensity and exposure time, and radially averaged to yield the scattering intensity I(q). The buffer curve was subsequently subtracted from the 4 sample curves. The resulting scattering curves were brought to absolute scale using water as a secondary standard^28^, taking into account the isothermal compressibility of water at 7 °C, Χ_T_ = 4.85 × 10^−10^ Pa^−1^. After verification that no concentration-dependent interparticle interference effects were present in the concentration-normalized scattering curves I(q)/c (Figure S1), they were averaged in order to improve the statistical quality of the final curve, which was used for further analysis. All data reduction was performed in the software RAW^29,30^. The MW of KDM5B was calculated from the concentration-normalized zero-angle scattering intensity I(0)/c on absolute scale^28^, taking into account the scattering contrast between the protein and the buffer. In preparation for the ATSAS-based *ab initio* modeling, the pair distance distribution function p(r) and the corresponding radius of gyration R_g_ and zero-angle scattering I(0)/c were calculated using the program GNOM^31^ through the ATSAS package^32^ integrated in RAW. 5 consecutive *ab initio* reconstructions were then performed using the program DAMMIF^33^. The runs were averaged by DAMAVER and filtered by DAMFILT^34^.

### Nucleosome pull-down experiments

Ni^2+^ precharged Protino Ni-TED resin (Macherey-Nagel) was used in the KDM5B and ccKDM5B His6 tag affinity pull-down experiments. KDM5B and ccKDM5B were used as bait whereas NCP were the prey. For the pull-downs with ccKDM5B a buffer with 25 mM HEPES, pH 7.7; 150 mM NaCl, 5% glycerol, 5 mM TCEP and 5 mM imidazole was used. For the pull-downs with KDM5B a buffer with 25 mM HEPES, pH 7.7; 300 mM NaCl, 5% glycerol, 5 mM TCEP and 5 mM imidazole was used. The same buffers were used for equilibration and washing of the resin. ~150 mg of Protino Ni-TED resin was added to an Eppendorf tube and 4 bed volumes of buffer were added to the resin and incubated in the rotation wheel at 4 °C for 1 hour in order to equilibrate. In the meantime, ccKDM5B and KDM5B aliquots were thawed on ice and buffer exchanged to its corresponding buffer using an Amicon 30 kDa ultra-0.5 mL centrifugal filter (Merck Millipore). The final concentrations of ccKDM5B and KDM5B were 3.2 mg/mL and 1.5 mg/mL, respectively. After ccKDM5B and KDM5B concentration and buffer exchange, 30 ul of both ccKDM5B and KDM5B in separate Eppendorf tubes were mixed with 2 ul of NCP (5.5 mg/mL) each. NCPs buffer consisted of 20 mM Tris, pH 7.5; 1 mM EDTA and 5 mM TCEP. Afterwards, 30 μL of the pre-equilibrated Protino resin were added to each Eppendorf and incubated on ice for 1 hour in order to let the complex bind to the resin. The tubes were centrifuged at 1000 rpm (Z 326 K, HERMLE Labortechnik) and the supernatant was saved for SDS-PAGE analysis (unbound fraction sample). The Protino resin was washed three times with 4 bed volumes using the corresponding buffer. The last washing fraction was saved for SDS-PAGE analysis (wash fraction sample). Finally, 30 ul of both ccKDM5B and KDM5B in separate Eppendorf tubes were mixed with 2 ul of NCP (5.5 mg/ml) each. A pull-down with only untagged NCPs was performed as a negative control.

### SPR measurements

SPR measurements were performed at 15 °C using a Pioneer FE instrument and the data were analyzed using the Qdat program version 2.6.3.0 (PALL FortéBio). KDM5B was immobilized by amine coupling on to a biosensor chip using a 10 mM NaAc pH 5 immobilization buffer. A HBS-EP running buffer (20 mM Hepes, 300 mM NaCl, 1 mM EDTA, 0.005% Tween 20, 1 mM DTT) was used for the experiments. The analytes were injected either in eight concentrations at 30 µL/min flow rate or in a gradient using the one-step injection at 150 µL/min flow rate over immobilized KDM5B. After each cycle, the surface was regenerated using 1 M NaCl. All sensorgrams were corrected for unspecific binding of the samples to the chip matrix and buffer effects by subtraction of blank and reference surfaces (the flow cell channel activated by injection of EDC/NHS and inactivated by injection of ethanolamine). The dissociation constants (K_D_) and maximum binding response (R_max_) were estimated with a reversible 1-step interaction model, assuming steady state at the end of analyte injection, using global non-linear regression analysis.

### Enzyme kinetics determination with the Formaldehyde Dehydrogenase (FDH) assay

The measurements were done in volumes of 25 µL dispensed in polystyrene NBS™ treated 384-well black flat-bottomed plates (Sigma). The enzyme concentration was 2.5 µM in all experiments. The assay buffer used was 50 mM HEPES pH 7.5, 50 mM NaCl, 50 µM FeSO_4_, 50 µM 2OG, 500 µM ascorbate, 2 mM NAD and 0.0125 U FDH. KDM5B was thawed on ice and subsequently diluted to 2.5 µM in the assay buffer and dispensed in 20 µL volumes in every second well of the 384 well plate. The plate was centrifuged at 1000 × g for a few minutes. Peptide dilution series were made using a stock of 5X the highest final concentration. For measurements with H3(1–21)K4me3, H3(1–15)K4me3, H3(1–10)K4me3, H3(1–8)K4me3 peptides (Caslo A/S and gifts from Novo Nordisk A/S) a 2-fold dilution series from 640 µM peptide was prepared. The reactions were started by adding 5 µL of substrate and then a Safire microplate reader (Tecan) was used to measure (excitation and emission wavelengths of 355 nm and 460 nm, respectively) the increase in fluorescence using 30 s scans for a minimum of 15 min. Samples without either KDM5B or peptide were included for each substrate and used for baseline correction and as negative controls. All data were converted to nM NADH formed per second using a NADH standard curve using the program GraphPad® Prism 5, and also fitted to a Michaelis Menten model written below (equation 1). As the substrate peptides can undergo more than one demethylation reaction^35^, true Michaels Menten kinetics will not be observed, and values of ${{K}_{{\rm{m}}}}^{app}$ are consequently reported. *k*_*cat*_ was determined using equation 2 (2). All assay measurements were carried out in triplicates and always repeated using two different protein preparations.

1 $${\rm{Y}}={V}_{{\rm{\max }}}\ast {\rm{X}}/({K}_{{\rm{m}}}+{\rm{X}})$$

2 $${k}_{cat}={V}_{{\rm{\max }}}/[{\rm{enzyme}}]$$

### HDX-MS measurements

6 pmol/ul KDM5B was prepared in 20 mM HEPES, pH 7.7 and 300 mM NaCl. HDX reactions of KDM5B samples were performed at RT for various time intervals from 15 s up to 24 h. Protein (6 pmol/uL) was diluted ten-fold into 99% D_2_O 20 mM HEPES pH 7.7, 300 mM NaCl to a final solution containing 90% D_2_O and 0.6 pmol/uL of KDM5B. Equilibrium-deuterated control samples were prepared in a 6M deuterated Gnd-HCl solution containing 90% D_2_O. Non-deuterated controls were made by dilution of samples into the same buffer without D_2_O. HDX reactions were quenched by a 1:1 (v/v) dilution into ice-cold buffer containing 6M Gnd-HCl, 300 mM Phosphate, pH 2.3. Quenched samples were frozen immediately and stored at a temperature of −80 °C. The samples were loaded onto a refrigerated UPLC system (nanoAcquity, Waters, Miliford, USA) and digested using an online pepsin column. Generated peptides were trapped and desalted on a C18 trap column (ACQUITY UPLC BEH C18 1.7 µm VanGuard column, Waters, Miliford, USA) for 3 min at 150 µL/min of 0.23% formic acid followed by separation on a C18 analytical column (ACQUITY UPLC BEH C18 1.7 µm 1.0 × 100 mm column, Waters, Miliford, USA) and eluted into a hybrid Q-TOF mass spectrometer (Waters Synapt G2si HDMS, Waters, Miliford, USA) using a 7 min gradient from 8% to 40% of 0.23% formic acid in acetonitrile at a flow-rate of 40 µL/min. MS was performed in positive ion mode, with internal mass-calibration using a reference lock-spray signal of Glu-Fibrinopeptide (Sigma-Aldrich, St. Louis, MO, USA). The peptides were identified using Data Independent Acquisition (MS^e^) Collision-Induced Dissociation (CID) MS/MS. The incorporation of deuterium was analyzed by DynamX ver. 3.0 (Waters, Miliford, USA) and visualized by PyMOL (The PyMOL Molecular Graphics System, Version 1.8 Schrödinger, LLC). The deuterium uptake of each peptide segment at each timepoint was normalized to the deuterium uptake of the equilibrium-deuterated control sample to account for differences in back-exchange of each peptide and thus provide an accurate measure of the absolute HDX of different regions of KDM5B^36^.

### Bioinformatics

Homology modelling was performed using the RaptorX server^37^. Prediction of coiled coil regions from the amino acid sequence was done with the COILS server^38^.

### EM data collection, 3D particle reconstruction and analysis

A diluted protein sample was added to a 400 mesh collodion and carbon coated copper grid that had been glow discharged and stained with 2% uranyl formate. Micrographs were collected at 120 kV using A Tecnai G2 Spirit TWIN electron microscope operated a 120 kV and a defocus of 0.7–1.7 µm were used for micrograph collection. Automated data collection employing Leginon^39^ and using a Tietz TemCam-F416 CMOS camera at a nominal magnification of 67,000x with a pixel size of 1.57 Å was performed.

The quality of the micrographs were inspected using XMIPP^40^; particle picking and 2D and 3D classifications were performed using the program Relion^41^, the initial 3D reconstruction was generated using the implemented Stochastic Gradient Descent algoritm and subsequently refined in Relion 2.1^41^. Initially, approximately 1000 particles were picked manually. After 2D class averaging into a total of 10 reference free class averages the 3 most populated classes were used for automated picking yielding a total of 135056 particles. After successive runs of 2D classification with a total of 300 to 100 classes where scarcely populated blurry or truncated protein classes were removed, the 7 most populated classes were selected for the generation of the initial model. The model was then refined by the same set of particles using Refine3D routine of Relion. This refine model was then used for 3D classification of well-defined protein particles into 5 classes. The most populated 3D class was then 3D auto refined using particles from the similar 3D classes. The final refine model was at a resolution of 27.1 Å. The fitting of the KDM5B 1–1078 model to the volume was done manually in the program Chimera^42^.

## Results

### Expression, purification and SEC characterization of KDM5B

The SEC trace (Figure S2A) shows a main peak eluting at a volume corresponding to about 2 times the MW of KDM5B. Before that, with close to baseline separation, a peak containing KDM5B and an additional band of MW around 55 kDa have eluted. The corresponding SDS-PAGE gel is shown in Figure S2B. To investigate the oligomeric state of KDM5B native gel electrophoresis of the main peak was undertaken (Figure S2C). Again, bands at around twice the MW of KDM5B and also at approximately four times the MW of KDM5B are observed. The equilibrium between these species was next investigated using analytical SEC of KDM5B from the central peak of Figure S2A in different concentrations that are relevant for biophysical studies (Figure S2D). In this range the position of the peak does not change, which shows that concentrating KDM5B samples to around 11 μM does not promote self-association significantly.

The high KDM5B MW determined from SEC and native gel electrophoresis can either be due to homo- oligomer formation or to an elongated shape of the molecule. To investigate this, we determined a KDM5B hydrodynamic radius R_H_^43^ of 77 Å from the SEC elution volume on a calibrated column (Figure S2E).

### Structural and flexibility information from SAXS data

**Figure 2:**
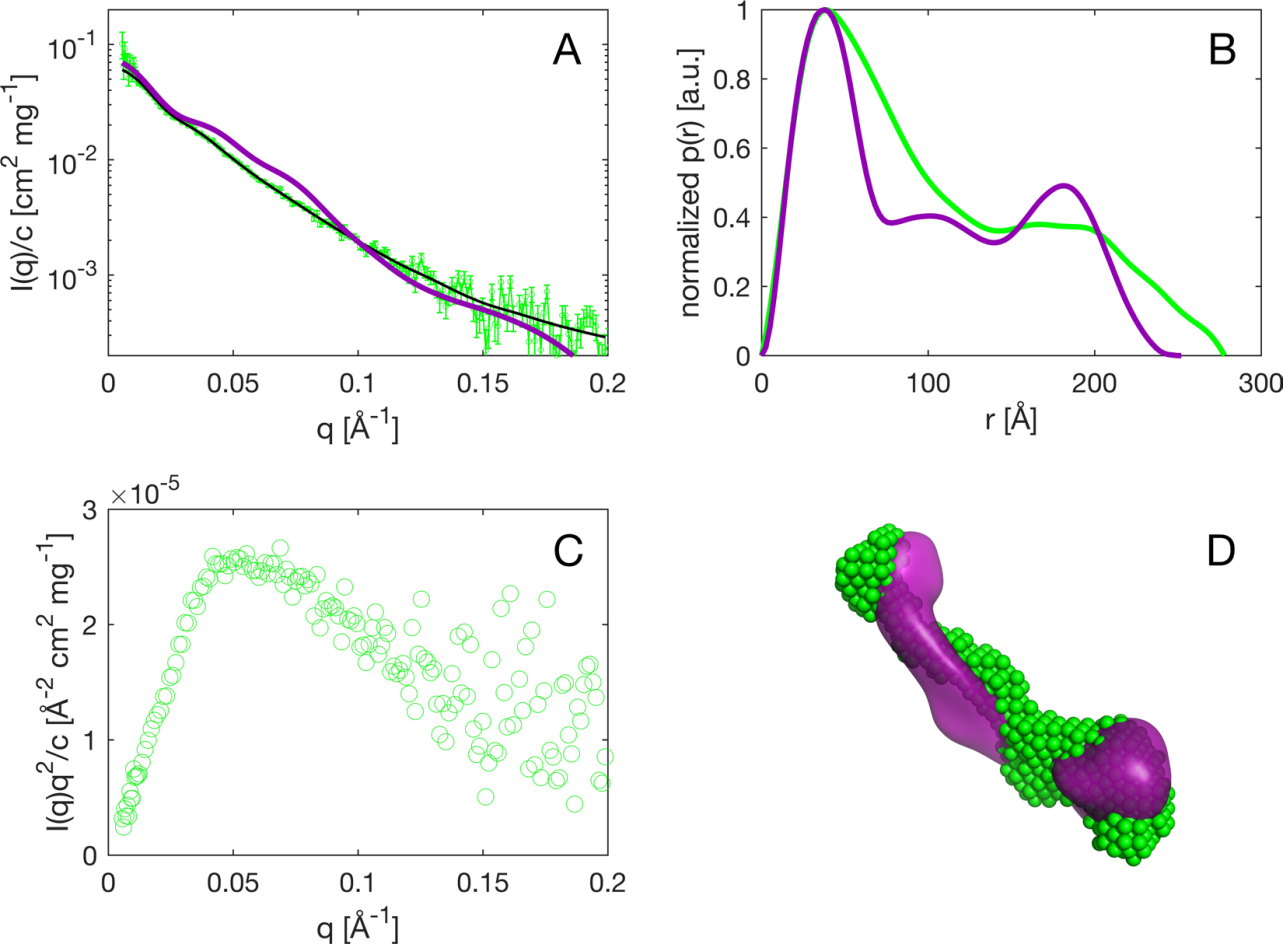
Solution SAXS of KDM5B. (**A**) SAXS curve of the measured KDM5B (green) with error bars corresponding to the experimental noise, the smooth I(q) generated from the inverse Fourier transform of the experimental data (black) and the calculated curve from the EM model of KDM5B (purple) using EM2DAM from the ATSAS suite^32^. (**B**) Pair distance distribution function p(r) of the measured KDM5B (green) and the calculated curve from the EM model (purple). (**C**) Kratky plot of the measured KDM5B. (**D**) *Ab initio* model of the measured data on KDM5B (green dots) overlaid with the EM model (purple surface).

The SAXS curve obtained for KDM5B is shown in Fig. 2A, with the corresponding pair distance distribution function p(r) in Fig. 2B. As the Guinier regime of a protein as large as KDM5B is barely accessible by SAXS, we additionally performed inverse Fourier transform analysis using GNOM^31^ in order to determine R_g_, I(0)/c and the longest extension of the particle, D_max_. The obtained R_g_ are (88 ± 33) Å from the Guinier approximation and (85 ± 2) Å from GNOM, and the derived R_H_/R_g_ ratio is 0.9, between the ratio of a sphere (0.775^44^) and that of a coil (1.2–1.6^45^). This indicates an elongated shape of the molecule. The maximum dimension, D_max_, of KDM5B is found to be 269 Å, demonstrating that it is a non-globular elongated molecule. The Kratky plot (Fig. 2C) suggests that KDM5B is an overall folded protein, and the pair distribution function p(r) (Fig. 2B) further indicates that the protein is dumbbell-shaped^46^. Based on the absolute scale and the scattering contrast between protein and buffer, the MW is calculated to be 152 kDa (Guinier) or 121 kDa (GNOM); differing somewhat from the value of monomeric KDM5B. However, based on the R_g_ and the calculated MW, it can be concluded that KDM5B, under the given buffer conditions, is primarily monomeric at a concentration of 10 μM and lower. Next, *ab initio* modeling was undertaken to obtain a low-resolution envelope of the solution structure of KDM5B. The filtered KDM5B bead model^47^ is shown in Fig. 2D, from which the molecule’s dumbbell shape is recognizable.

### Kinetic characterization of KDM5B enzymatic activity

To investigate whether the produced recombinant KDM5B is catalytically active, four substrate mimicking peptides were tested in the FDH coupled assay^48^ (Table 1 and Figure S3). The ${{{\rm{K}}}_{{\rm{m}}}}^{{\rm{app}}}$ values are seen to be in the low μM range, with the 10-meric peptide as an exception. This particular length of peptide may introduce non-compensated strain in the peptide conformation e.g. interactions of a side chain in residues residing in the peptide terminus that are lost upon peptide length extension. The ${{\rm{K}}}_{{\rm{cat}}}/{{{\rm{K}}}_{{\rm{m}}}}^{{\rm{app}}}$ values are seen to be clearly higher for the longer peptides.

**Table 1:** Substrate kinetic parameters.

Substrate peptide	H3(1–8)K4me3	H3(1–10)K4me3	H3(1–15)K4me3	H3(1–21)K4me3
K_cat_ *10^4^ (s^−1^)	51.9 ± 2.5	57.9 ± 3.8	35.7 ± 1.7	29.1 ± 2.7
${{{\rm{K}}}_{{\rm{m}}}}^{{\rm{app}}}$ (µM)	4.2 ± 0.8	19.1 ± 3.8	1.0 ± 0.3	1.1 ± 0.5
**95% Confidence Intervals**				
K_cat_ *10^4^ (s^−1^)	46.7 to 57.1	50.0 to 66.0	32.1 to 39.3	23.4 to 34.8
${{{\rm{K}}}_{{\rm{m}}}}^{{\rm{app}}}$ (µM)	2.6 to 5.8	11.2 to 27.0	0.4 to 1.6	0.2 to 2.1
R²	0.9283	0.9251	0.8525	0.7098
${{\rm{K}}}_{{\rm{cat}}}/{{{\rm{K}}}_{{\rm{m}}}}^{{\rm{app}}}$ *10^4^ (s^−1^/µM)	12.4	3.0	36.1	25.3

### KDM5B and NCP interaction

**Figure 3:**
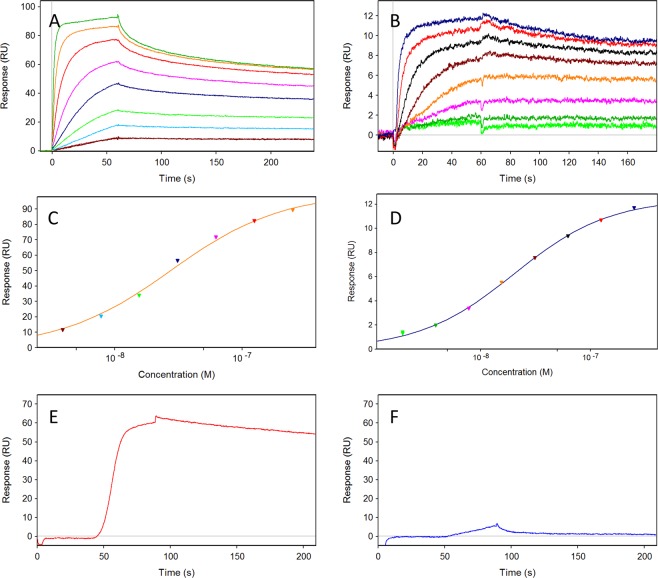
Interactions between KDM5B and NCPS demonstrated in real-time by SPR biosensor technology. 3.9 to 250 nM NCPs injected over 975 RU (**A**) or 490 RU (**B**) immobilized KDM5B. (**C**,**D**) Steady state analyses of sensorgrams in A-B were based on report points taken at the end of the injection, although steady state was not reached in all report points. (**E**,**F**) Gradient one-step injection of up to 500 nM of the positive control NB8 (**E**) and the negative control NB17 (**F**) over 881 RU immobilized KDM5B. All experiments with injections of NCPs over immobilized KDM5B included successful regeneration of the surface between injections with 1 M NaCl. All sensorgrams are blank injection and reference surface subtracted.

In order to validate the interactions between recombinant KDM5B and NCPs, pull-down experiments were conducted (Figure S4). As shown in Figure S4 intact recombinant KDM5B can pull-down recombinant unmodified nucleosomes. To quantify, SPR was applied. NCPs were injected in eight concentrations (two-fold serial dilution ranging from 250 nM to 1.91 nM) over immobilized KDM5B (975 RU and 490 RU) (Fig. 3A,B). The experiments showed a stable complex formation between KDM5B and NCPs, with slow dissociation rate. The interaction between KDM5B and NCPs had to be abrogated using a high ionic strength (1 M NaCl) regeneration solution, so that the signal returned to baseline before a new concentration of KDM5B could be injected. From steady state affinity analysis of the SPR sensorgrams (Fig. 3C,D), the dissociation constants (K_D_) were determined to be in the lower nM range (Table 2). As the complexity of interaction mechanism may not be compatible with a 1:1 interaction, the steady state K_D_-values are termed “apparent” (${{{\rm{K}}}_{{\rm{D}}}}^{{\rm{app}}}$).

**Table 2:** Steady state data for the interaction between NCPs and immobilized KDM5B. The parameters were determined using a 1:1 steady state affinity model.

Surface	Data analysis	KD (nM)	Rmax (RU)
KDM5B (490 RU)	Steady state	20.3 ± 0.1*	12.48 ± 0.02
KDM5B (975 RU)	Steady state	27.7 ± 0.1*	100.6 ± 0.1

Data from Fig. 3A–D. *The interaction mechanism is not established to be a 1:1 interacton and the estimated KD-values should be termed “apparent” (${{{\rm{K}}}_{{\rm{D}}}}^{{\rm{app}}}$).

We have previously raised nanobodies against KDM5B^22^. One of these (NB8) was shown to bind strongly in SEC experiments whereas another (NB17) did not bind at all. To verify the structural integrity of the immobilized KDM5B, the positive control NB8 and the negative control NB17 were injected in a one-step gradient over immobilized KDM5B (881 RU) (Fig. 3E,F). NB8 showed a very strong binding to the KDM5B, with a slow off-rate. The binding was unbreakable using 1 M NaCl as regeneration solution and any suitable condition to break the interaction for regeneration of the immobilized surface was not found, wherefore experiments with multiple cycles were not possible. In comparison, NB17 did not show any specific binding to KDM5B. The gradient one-step injection of NB8 was fitted to a 1-1 interaction model deriving an approximate affinity of binding to KDM5B in higher pM range (Table S3).

### HDX-MS analysis of KDM5B

**Figure 4:**
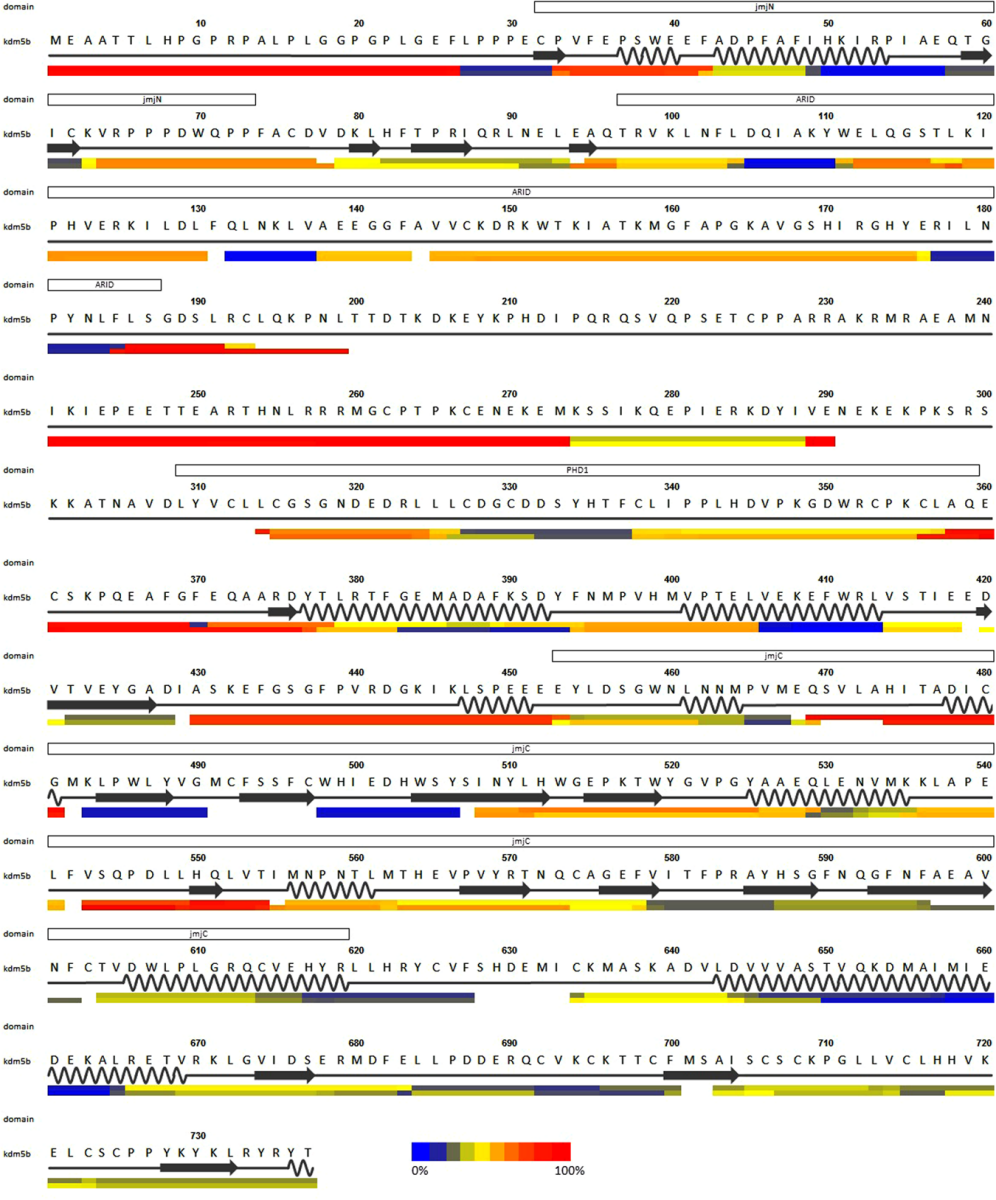
HDX-MS heatmap of KDM5B residues 1–739. Top line above the numbering show domain regions in blue. Second line shows the amino acid sequence. Third line shows the DSSP^56^ derived secondary structural elements from the structure of KDM5B residues 1–760 (PDBID 5A1F)^19^ with α-helices in black and β-strands in blue. The fourth and the fifth lines show HDX-MS 15 seconds exchange heat maps for KDM5B and ccKDM5B, respectively. bars in the heatmap are colored according to the extent of HDX normalized to the equilibrium-labeled control. The color gradient for normalized deuterium exchange is shown in the bottom of the figure. Very short segments arise from differences of peptide lengths.

HDX-MS measures the hydrogen/deuterium exchange (HDX) of backbone amide hydrogens in a protein by use of mass spectrometry (MS). The rate of HDX of protein backbone amides reports on the presence and strength of local hydrogen bonding in the protein, with unstructured dynamic regions exhibiting fast HDX (subsecond to second timescales) and regions with higher-order structure containing transient or stable backbone hydrogen bonding exhibiting slower HDX (on a timescale from minutes to days). In order to gain information on the local conformational properties of KDM5B in solution, HDX-MS analyses were performed on the full length protein. HDX-MS analysis of KDM5B resulted in 313 identified peptic peptides that were used to resolve the local deuterium uptake of 87.3% of the full length protein sequence. An overview of the full HDX time course of KDM5B is shown as a heat map in Figure S5 and a representative uptake curve is shown in Figure S6. The 15 second HDX of individual regions of KDM5B 1–740 is mapped onto the sequence in Fig. 4, row 4. The HDX data clearly show that the first 27 residues are disordered with corresponding fast HDX, whereas significantly slower HDX is observed in the following jmjN and ARID domain regions. The following region (residues 198–375) is characterized by fast HDX and two regions that lack HDX data. However, the PHD1 domain region is characterized by slow HDX, demonstrating the presence of higher-order structure. In the jmjC domain, the segment containing res. 430–453 to seen to display very fast HDX. This agrees well with the KDM5B 1–753 crystal structure^19^, where the region 442–447 lacked electron density and was omitted from the model. The region 469–481 also shows fast HDX. As seen in the crystal structure, in particular the region around H474 is suggested to be disordered. The fast exchange of the region 543–554 also agrees with the poorly defined electron density around residue 545 in the crystal structure. In contrast, the surface exposed loop region (peptide 563–574) of ccKDM5B exchange quickly with a dynamic conformation in solution but has a very well defined electron density in the crystal structures. The entire region comprising residues 575–740 show only slow to moderate HDX; this indicates the presence of many secondary structural elements. This is in good agreement with the 5A1F crystal structure that revealed an α-helical region (residues 604–671 and 737–753), a β-sheet composed of three β-strands (residues 673–734) that harbor the C5HC5 zinc finger motif and an α-helix (residues 736–751).

**Figure 5:**
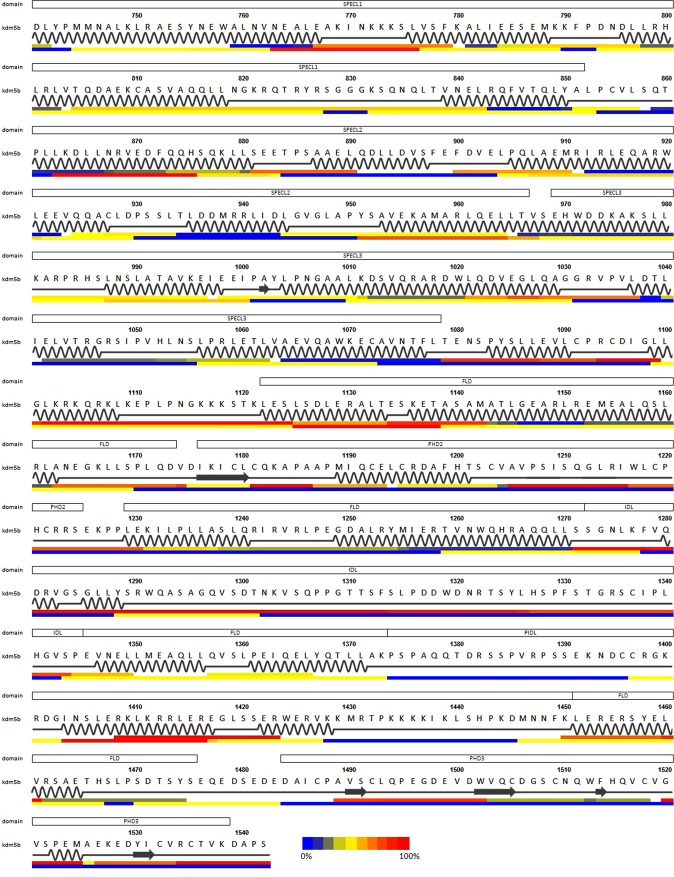
HDX-MS heatmap of KDM5B residues 740–1544. Top line above the numbering show annotated domains. The second line show the amino acid sequence numbers with the PLU region 768–1100 boxed. Third line shows the amino acid sequence. Fourth line shows the sequence-only derived secondary structure by the RaptorX server with α-helices represented by curvy line and β-strands represented by arrows. The fifth line shows the HDX-MS 15 seconds exchange heat map for KDM5B with the same coloring scheme as in Fig. 1. Sixth line shows the predicted coiled coil propensity using the COILS server with same color gradient as for the HDX-MS data with color mapping red as high propensity and blue low.

The HDX-MS heat map of the structurally hitherto uncharacterized KDM5B residues 753–1544 is shown separately in Fig. 5, row 5. Remarkably many helices are predicted following the last residue 753 in the crystal structure and all the way through the PLU region (boxed sequence). Also, all of the predicted helices are characterized by protection from HDX, strongly supporting the presence of secondary structural elements. Also, three coiled coil motifs are predicted in HDX protected regions (763–777, 862–876 and 952–967), notably separated by approximately 100 residues. A weak coil-coil signature is also observed approximately 100 amino acids downstream of these 3 motifs, at residues 1056–1072. After this the region 1078–1125 displays fast HDX. Next follows a region 1143–1174 exhibiting protection from HDX, predicted to be a helical structure. This region flanks the PHD2 domain. Another predicted helical region 1228–1270, supported by the HDX data, is located immediately after the PHD2 domain, again followed by a long unstructured region 1271–1344 with fast HDX. This region is followed by another predicted helical region 1345–1365 with reduced HDX that in turn is followed by a region 1376–1450 with sparse HDX sequence coverage. Data for the only peptide from this region shows fast HDX demonstrating a disordered structure despite a predicted helicity. Just before the terminal PHD3 domain another region exhibiting reduced HDX showing the presence of structure, predicted to be helical. In good agreement with the sequence annotation, both of the annotated PHD2 and PHD3 domains have regions with slow HDX, corresponding to stable higher-order structure.

### Comparative HDX-MS analysis of KDM5B to ccKDM5B

**Figure 6:**
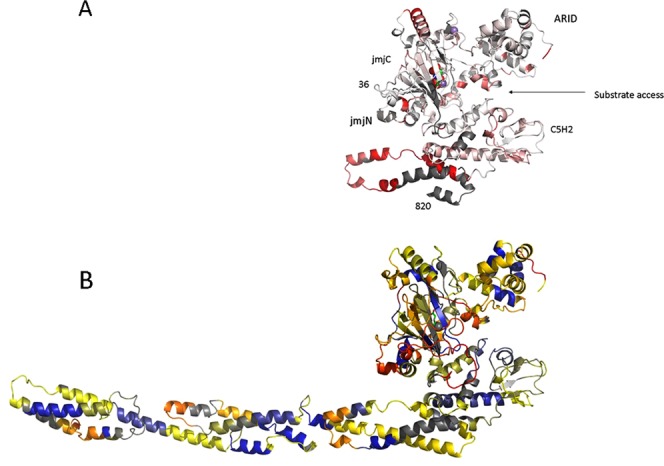
(**A**) Mapping of differences in HDX-MS data between ccKDM5B and KDM5B using a white to red coloring scheme mapped on to a KDM5B 1–820 homology model. Color mapping from no difference in exchange between ccKDM5B and KDM5B colored white to large differences in red. First and last visible residues in the figure are labeled. Domains and substrate access direction are also indicated. (**B**). Mapping of HDX-MS data to the model of KDM5B 1–1078 using a blue-yellow-red coloring scheme. Regions 1–24 and 196–378 are omitted as no reliable templates are available. Regions where no exchange data is available is colored grey.

In order to analyze if the N-terminal and the C-terminal regions of KDM5B have a conformational impact on each other through intra-molecular interactions, HDX-MS experiments was also undertaken of ccKDM5B (Fig. 4, row 5). Regions with significant differences in HDX between the two molecules are mapped on the structure of ccKDM5B in Fig. 6A. The largest differences in HDX are observed in the regions 392–395 and 761–804.

### Homology modeling of KDM5B

Homology modeling was next undertaken to further investigate the 3-dimensional structure of KDM5B. Based on sequence similarities the RaptorX server identified and used the following templates: Initially a model of residues 1–760 was build based on the crystal structures of ccKDM5B and KDM5A residues 1–785^20^ (PDBID 5CEH). KDM5A residues 1–785 is highly homologous to ccKDM5B and also includes the ARID domain. Next a model of region 736–1270 was build. The model for the first 348 residues (736–1078) was built from the crystal structures of the rod domain of human α-actinin^49^ (PDBID 1HCI) and the crystal structure of the human muscle α-actinin-2^50^ (PDBID 4D1E). Both structures are dimers of 4 spectrin domains where the KDM5B modeling was based on spectrin domains 2–4. The P-value for the modeling of this part of the structure was reported as 1.e-4, clearly below the reported threshold for a reliable model. In contrast, it was not possible to build reliable models neither for the sequence region 1085–1270 nor of the entire 1085–1544 region. By superimposing the helical regions 741–749 from the models of regions 1–760 and 736–1078 using PyMol, a final 1–1078 N-terminal model of KDM5B was constructed. Figure 6B show the KDM5B HDX-MS data mapped on the structure of KDM5B 1–1078.

### EM analysis of KDM5B

**Figure 7:**
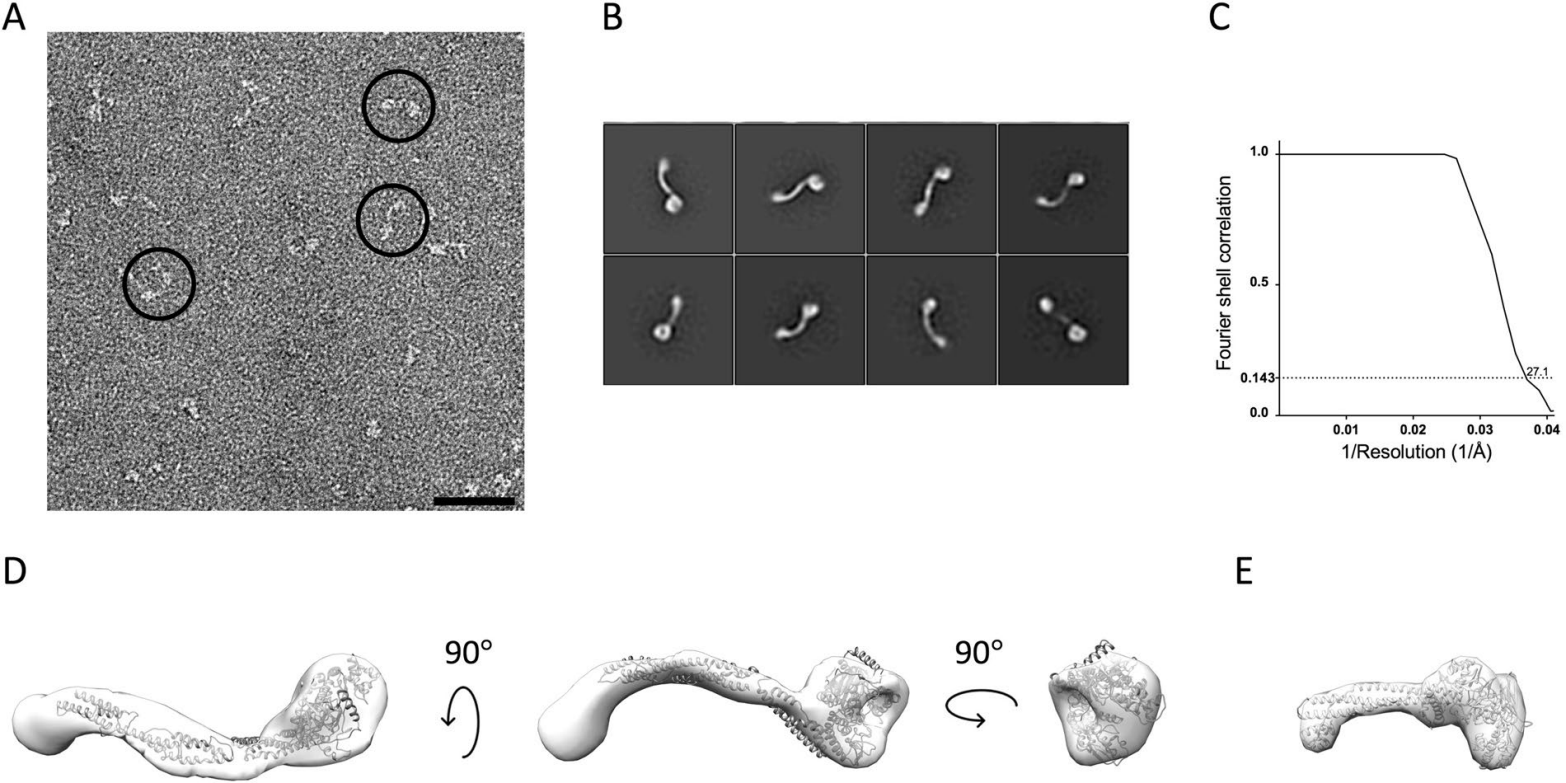
KDM5B EM data. (**A**) A representative micrograph with particles (black bar measures 500 Å). (**B**) The 8 most populated EM reference free 2D class averages (box edges measure 567 Å). The most populated class is in the upper left corner. (**C**) FSC curve from the refinement of the particle. (**D**) Fitting of KDM5B 1–1078 to the derived 27 Å KDM5B volume in three different orientations. (**E**) 27 Å resolution volume calculated of the KDM1A/CoREST complex.

In order to further validate the model based on SAXS, HDX-MS and homology modeling negative stain EM experiments were undertaken. Figure 7A show an excerpt from a micrograph with characteristic particles circled; Fig. 7B the 25 most populated reference free 2D-class averages. The classes clearly suggest that the structure of KDM5B comprises two globular domains separated by a linear narrower linker. The larger of the two domains appears more well-defined than the smaller. As the catalytic domain is the largest moiety of KDM5B, this is in agreement with a higher content of intrinsically disordered loop regions in the C-terminal part of the molecule. Figure 7C shows the Fourier Shell Correlation^51^ (FSC) curve after the final refinement cycle suggests a resolution of 27 Å. Figure 7D display the fitting of the KDM5B 1–1078 homology model to the derived 3D volume. The individual SPECL domains were fitted manually with respect to each other to fit the curved shape of the derived volume. Despite the helix prediction algorithm and that the HDX-MS data suggests that a C-terminal domain is present in the structure, no fitting of the region 1079–1544 was attempted due to the limited resolution.

## Discussion

SEC experiments suggest that KDM5B is either a dimer in solution or that it is an elongated molecule (Figure S2A–D). From the SAXS data (Fig. 2) it can be concluded, that KDM5B is essentially monomeric in solution and that it exhibits an elongated shape with a dumbbell-like architecture. The KDM5B R_H_ values derived from a SEC calibration curve (Figure S2E), the R_g_ from the SAXS experiment and also the R_H_ derived from the volume originating from the EM characterization discussed below are all consistent with an elongated shape of the molecule. For concentrations relevant for EM characterization (below 11 μM) no significant oligomerization is observed (Figure S2C).

The kinetic characterization using substrate mimicking peptides revealed KDM5B properties similar to those of other HDMs^24^ (Table 1). As for ccKDM5B, higher substrate specificity is achieved with the longer peptide. The values of ${{{\rm{k}}}_{{\rm{m}}}}^{{\rm{app}}}/{{\rm{k}}}_{{\rm{cat}}}$ for the full length enzyme are generally comparable to those of ccKDM5B^24^.

The pull down experiments with recombinant KDM5B (Figure S4) show that the full length enzyme bind NCPs in sub-stoichiometric amounts. In contrast, the N-terminal ccKDM5B fragment does not appear to bind NCPs despite the presence of the PHD1 domain with established affinity for H3K4me0^17,18^. This is, however, in agreement with earlier findings^21^, that showed that the KDM5B C-terminus, but not the N-terminus, can pull down nucleosomes isolated from HeLa cells. Taken together, this suggests that the nucleosome interaction is mediated either by the helical domain or by the following C-terminal region. The PHD3 domain can be excluded, as the above mentioned studies of KDM5B PHD domain specificities mapped this domains specificity to H3K4me3. The SPR experiments (Fig. 3) confirm the direct binding between KDM5B and the nucleosomes. The interaction is strong, with a very slow dissociation and a ${{{\rm{K}}}_{{\rm{D}}}}^{{\rm{app}}}$ in low nM range (Fig. 3 and Table 2). This is an almost 100-fold stronger affinity compared to what is observed for KMD1A:CoREST using nucleosomes with the same Widom 611 DNA sequence in an HI-FI (high-throughput interactions by fluorescence intensity) assay, and 10-fold stronger than what is observed for the same complex when the DNA sequence is extended with extra-nucleosomal DNA in the same assay^52^.

The HDX-MS heat map for residues 1–739 (Fig. 4) show a generally good agreement between the experimentally determined HDX profiles and the X-ray structure. Residues 102–373 are absent in the ccKDM5B 5A1F crystal structure. Residues 201–240 are also not recovered in the HDX-MS experiment. For the rest of this region the data suggest that the region consists of two disordered regions 185–200 and 251–275 that are flanking a slow exchanging region that includes the residues 306–360 PHD1 domain as determined by NMR spectroscopy^18^ and the region 275–298. This further could suggest that the entire region 276–360 form an extended PHD domain.

The HDX-MS heat map of KDM5B residues 740–1544 (Fig. 5) shows that here the protein contains abundant regions with stable backbone hydrogen bonding indicative of several secondary structural elements. The presence of three regions of length around 100 residues with reduced HDX and predicted central coiled-coil structure could suggest an actinine like structure in this region, consequently with an elongated rigid structure consisting of 3 spectrin-like (SPECL1-3) repeats^53,54^. The presence of spectrin domains in non-structural proteins is not uncommon, they have for example been found in other multi-domain proteins comprising for example Rho-GEF, PH and SH3 domains^53^.

The region between the putative SPECL3 domain and the terminal PHD3 domain is characterized by a flexible linker and four helical regions separated by the PHD2, an intrinsically disordered region and a region with low HDX data coverage. From the latter region, HDX was only mapped for one peptide segment. As this segment comprise the only predicted helix and show rapid HDX, our data suggest that this entire region is intrinsically disordered as well. Overall, this architecture could suggest the presence of a flexible loop containing helical domain (FLD) with three large loop regions, one with only a short flexible linker containing the PHD2, one containing an intrinsically disordered loop (IDL) and one containing a putative intrinsically disordered loop (PIDL). As the linker to the C-terminal PHD3 domain is as short as the PHD2 linker, both of these domains must be in close proximity to FLD.

The major differences in HDX between ccKDM5B and KDM5B are found in the C-terminal region of ccKDM5B (Fig. 6A). It is likely that these differences arise from the truncation made in ccKDM5B, as this region in the native full-length protein exhibits local HDX corresponding to higher-order structure. Thus, the comparative HDX-MS analysis shows that the N- and C-terminal parts of KDM5B do not exert major conformational impacts on each other and if interactions occur between these two halves, they are transient or dynamic in nature.

During modeling of a large number of truncations of KDM5B that comprise the PLU region, the alignment algorithm of the RaptorX server consistently identified actin molecules as templates for homology modelling. The HDX-MS data is a valuable validation source of the constructed models in this context. Remarkably, Fig. 6B with the mapping of the HDX-MS data on the model of the region show good agreement between the spectrin helical sections and slow HDX. A schematic representation of a hypothetical domain structure of KDM5B, including the FLD, is shown in Fig. 1. The FLD could not be modeled by RaptorX so interactions and the relative directionalities of the helices are undetermined from the HDX experiments only; consequently the helices are just drawn in a sequential manner.

The reference free 2D class averages derived from the EM experiments (Fig. 7B) suggest that KDM5B particles corresponding to essentially only one abundant conformation have been picked from the micrographs. The resolution obtained is 27 Å and there are a limited number of preferred orientations observed on the micrographs. Attempts to do 3D classifications resulted in just one 3D volume, shown in Fig. 7D after fitting of the 32–1078 structure. The derived volume supports the presence of the three SPECL domains and the FLD domain. It is also in overall good agreement with the bead model derived from the SAXS experiments (Fig. 2D). However, the envelope derived from the SAXS data is longer and more linear, with a relatively less pronounced dumbbell-like shape. This is also reflected in the differences between the measured and calculated scattering curves (Fig. 2A) and the derived p(r) functions (Fig. 2B). These significant differences could be explained by the assumption that the template-based picking procedure has selected one conformation only, whereas the SAXS data gives an average of all conformations in solution. This further implicates that KDM5B is quite dynamic in solution as is also reflected by the high NSD value (Table S2). Other explanations of the observed differences, based on experimental differences between the two techniques, are, however, also possible. It is, however, very notable that the curvature of the EM derived conformation (Fig. 7D) of KDM5B appears to be complementary to the overall shape of mono-nucleosomes as determined from the crystal structure^55^.

This study is the first determination of the overall 3-dimensional architecture of one of the 30 Jumonji family histone demethylases encoded in the human genome^10^. Due to the high degree of similarity in primary sequence and domain organization, it is likely that the other members of the KDM5 subfamily KDM5A, KDM5C and KDM5D have a similar architecture. The structure consequently lays the foundation for new hypotheses about the function on the molecular level of this important family of enzymes.

The derived KDM5B volumes have striking architectural similarities to a low resolution volume of the KDM1A/CoREST complex (Fig. 7E). Both enzymes have a structure with two domains separated by a linear linker region. In both molecules, there is an N-terminal domain with eraser functionality and a C-terminal domain with reader functionality. The major differences are the length of the linker region and the complexity of the reader domain. The linker in KDM5B is, however, much longer than that of KDM1A. Also, where the C-terminal SANT domain of CoREST is only known to bind unspecific DNA sequences, KDM5B here comprise two PHD domains and large loops that may mediate protein-protein interactions.

In conclusion, in light of these structural similarities it is tempting to speculate, that there are also functional similarities between KDM5B and the KDM1A/CoREST complex. In particular; is the FLD domain initially scanning the nucleosomal DNA surface before demethylation can occur in the same manner as the KDM1A/CoREST SANT domain. For further investigation, structural studies of the interaction between nucleosomes and KDM5 enzymes are required.

## Supplementary information

Supplementary material
