# Supplementary material for: Molecular architecture of the Jumonji C family histone demethylase KDM5B
Source: Sci Rep. 2019 Mar 11;9:4019. doi: 10.1038/s41598-019-40573-y (PMC6411775; doi:10.1038/s41598-019-40573-y)
Supplement: Supplementary file 1 — Supplementary material [file 41598_2019_40573_MOESM1_ESM.docx]

**Molecular architecture of the Jumonji C histone demethylase KDM5B**

Jerzy Dorosz^1^, Line Hyltoft Kristensen^1^, Nanda G. Aduri^1^, Osman Mirza^1^, Rikke Lousen^1^, Saskia Bucciarelli^1^, Ved Mehta^1^, Selene Sellés-Baiget ^1^, Sara Marie Øie Solbak^2^, Anders Bach^2^, Pablo Mesa^3^, Pablo Alcon Hernandez^3^, Guillermo Montoya^3^, Tam T. T. N. Nguyen^4^,Kasper D. Rand^4^,Thomas Boesen^5^and Michael Gajhede^1^

^1^Biostructural Research, Department of Drug Design and Pharmacology, Faculty of Health and Medical Sciences, University of Copenhagen, Jagtvej 162, 2100 Copenhagen, Denmark

^2^Medicinal Chemistry, Department of Drug Design and Pharmacology, Faculty of Health and Medical Sciences, University of Copenhagen, Jagtvej 162, 2100 Copenhagen, Denmark

^3^Protein Structure & Function Programme, Macromolecular Crystallography Group, Novo Nordisk Foundation Center for Protein Research, Faculty of Health and Medical Sciences, University of Copenhagen, Blegdamsvej 3B, Copenhagen 2200, Denmark

^4^Department of Pharmacy, Faculty of Health and Medical Sciences, University of Copenhagen, Universitetsparken 2, 2100 Copenhagen, Denmark

^5^Interdisciplinary Nanoscience Center, Aarhus University, Gustav Wieds Vej 14, 8000 Aarhus, Denmark

* Correspondence: Michael Gajhede [mig@sund.ku.dk](mailto:mig@sund.ku.dk)

Running title: Molecular architecture of KDM5B

Keywords: Histone demethylase, nucleosomes, hydrogen deuterium exchange mass spectrometry, negative stain electron microscopy, surface plasmon resonance

**Figure captions**

Figure S1: Comparison of solution SAXS data of KDM5B at 4 different concentrations (0.75, 1, 1.5 and 1.8 mg/ml). A: Concentration-normalized scattering intensity. B: Ratio of scattering intensities. C: Kratky plot.

Figure S2: KDM5B SEC, SDS/PAGE and native PAGE. A. 280 nm UV trace from size exclusion chromatography of KDM5B using a HiLoad 26/600 Superdex 200 pg column. The column void is indicated with a vertical black bar. B. SDS-PAGE analysis of the marked fractions from A. C. Analytical SEC of KDM5B in concentration range 0.05-2 mg/ml. D. 3-12% Blue Bis-tris NativePAGE gel (ThermoFisher, Life Technologies) with 1.5, 0.75 μg KDM5B and markers loaded. Destained and subsequently stained with Silver. E. Superdex 200 5/150 Increase (GE Healthcare) calibration curve. Elutions of standards, KDM5B and that calculated from the EM model of KDM5B calculated using programs vol2pdb ^1^ and hydropro ^2^ are indicated.

Figure S3. Kinetic characterization of KDM5B using the FDH assay A: Michaelis Menten curves 0-120 μM substrate concentration. B: Michaelis Menten curves zoom 0-16 μM substrate concentration.

Figure S4: KDM5B: Nucleosomes pull-down experiments. Gel to the right: SDS-PAGE analysis of three pull-down experiments: NCPs pull-down (U: unbound, W: wash and B: bound), KDM5B/NCPs complex (U: unbound, W: wash and B: bound) and BR305/NCPs complex (U: unbound, W: wash and B: bound). Separate gel to the left: A positive control for NCPs, KDM5B and ccKDM5B were added. ). M is used as a Mw standard. Mw of the standards is placed next to its band and stated in kDa.

Figure S5. Full time course of KDM5B HDX data. Timepoints 0.25, 1, 10, 60 and 1440 minutes. Sequence numbering starts at -2 as 3C protease cleavage leaves 2 N-terminal residues from the linker.

Figure S6. Representative protein region displaying slow HDX. A. HDX as a function of time for region 49-77 IHKIRPIAEQTGICKVRPPPDWQPPFACD. B. MS-spectrum of +4 ion at time 0. C. MS-spectrum of +4 ion after 15 seconds. Blue lines show calculated spectra.

**Table captions**

Table S1. Details of SAXS data collection.

Table S2. Details of SAXS data analysis.

Table S3. Kinetic data for the interaction between NB8 and immobilized KDM5B. The parameters were determined using a fit to a 1:1 Langmuir model.

**Figure S1**

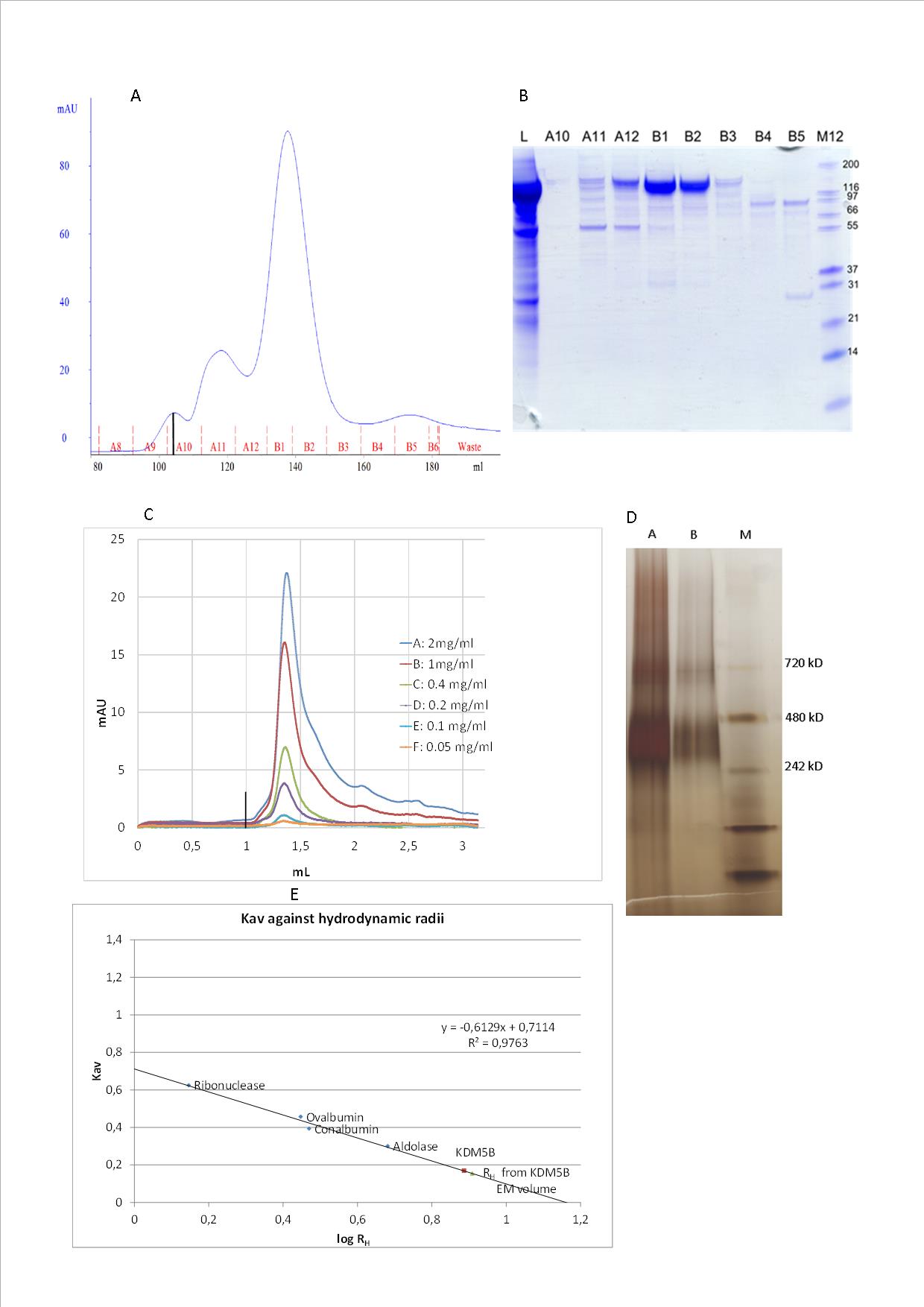
**Figure S2Figure S3**


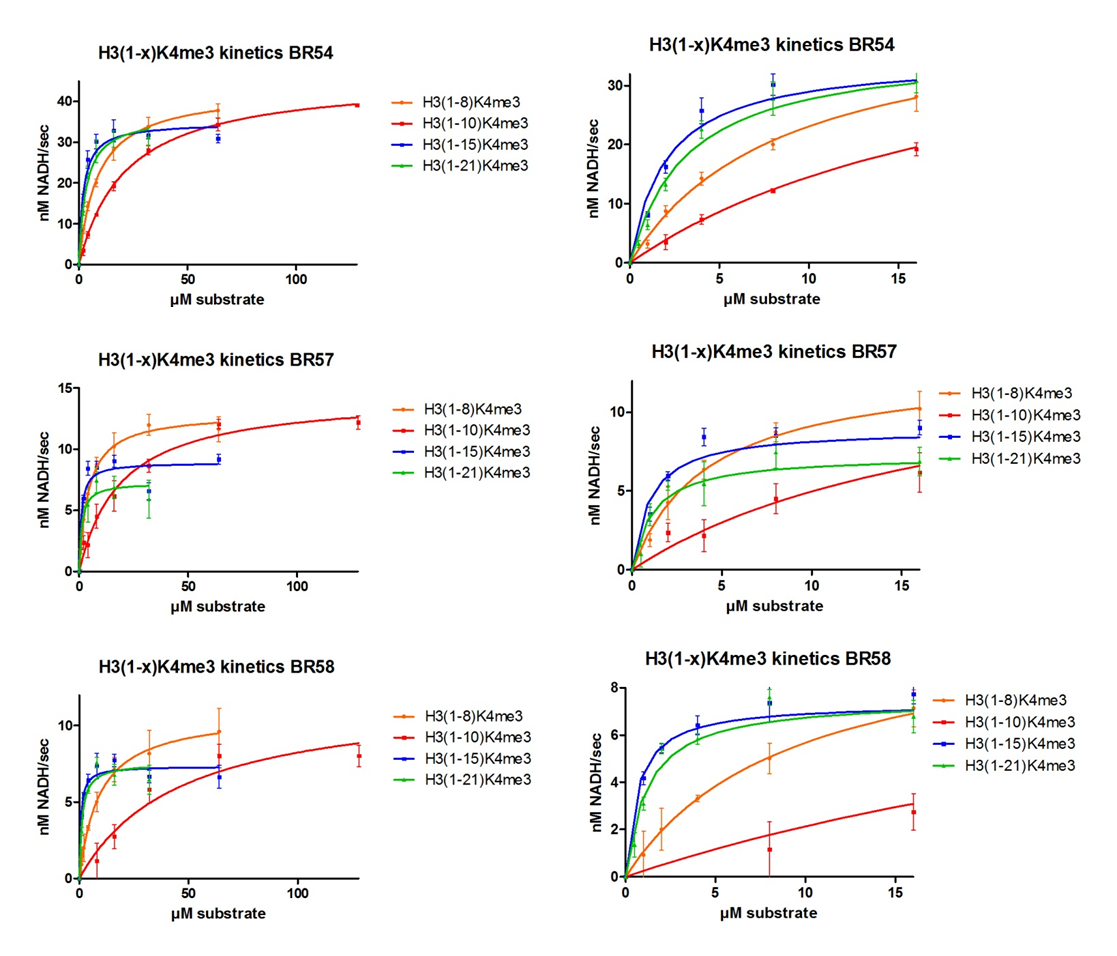


**Figure S4**


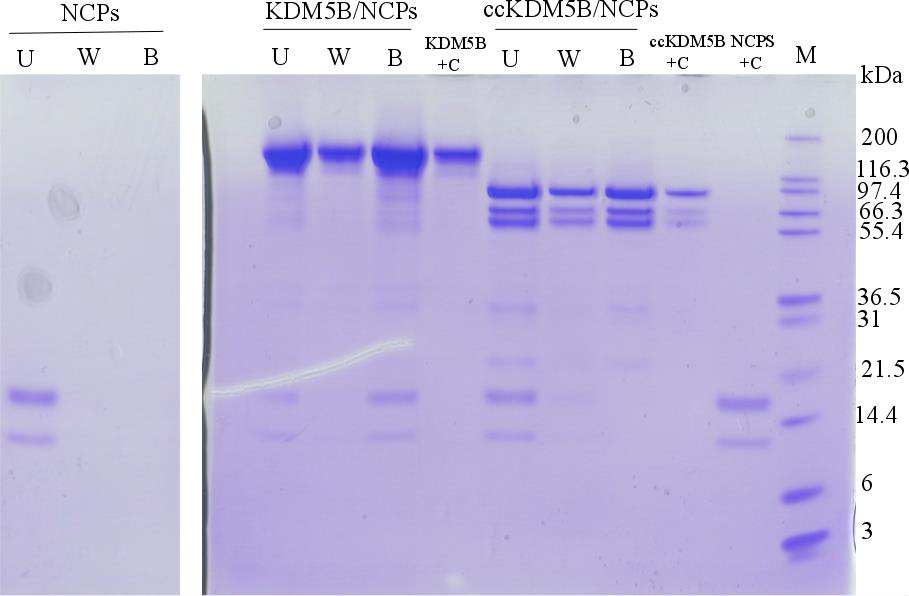


**Figure S5**


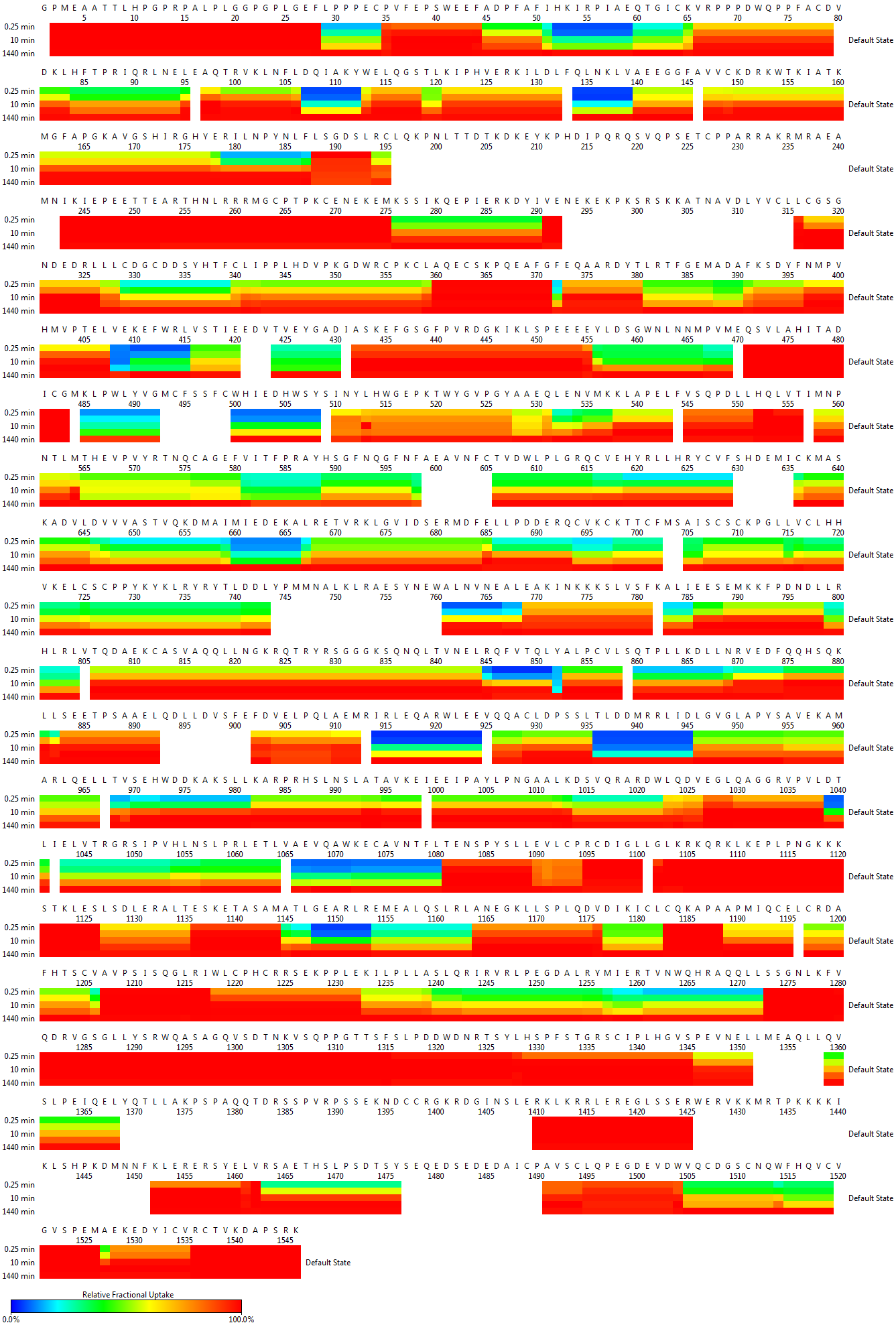


**Figure S6**

A


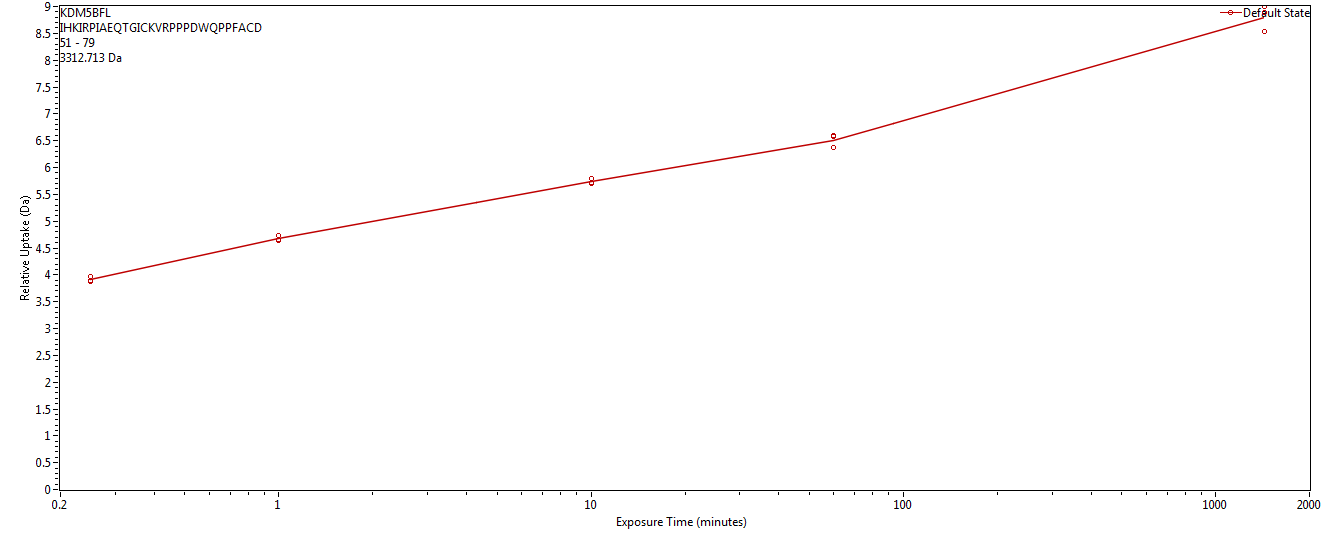


B


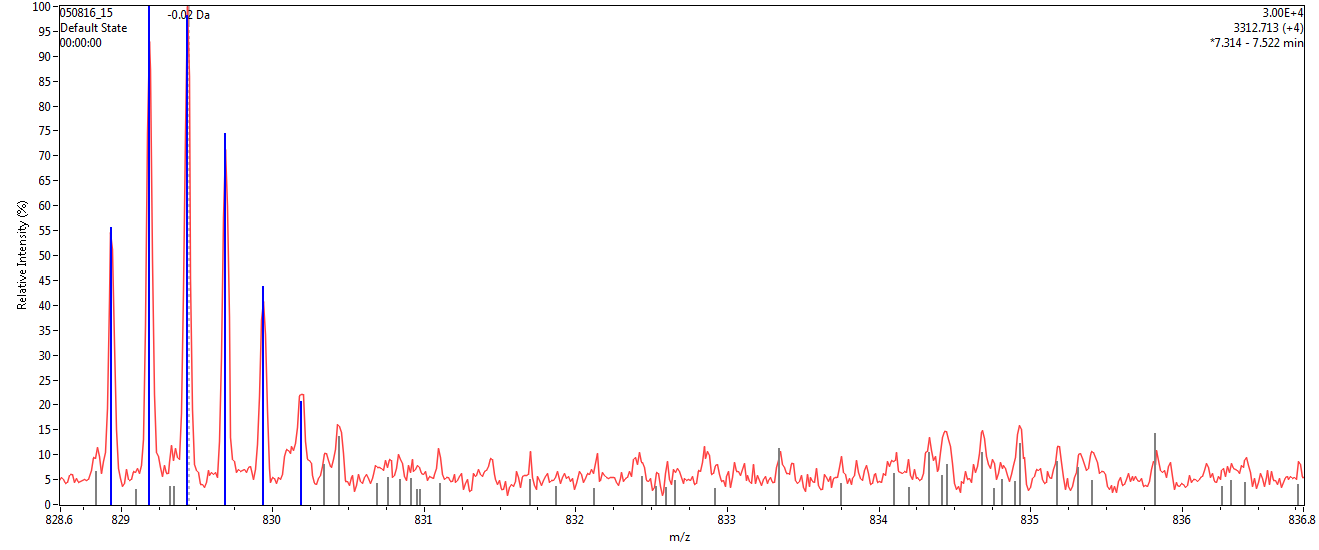


C


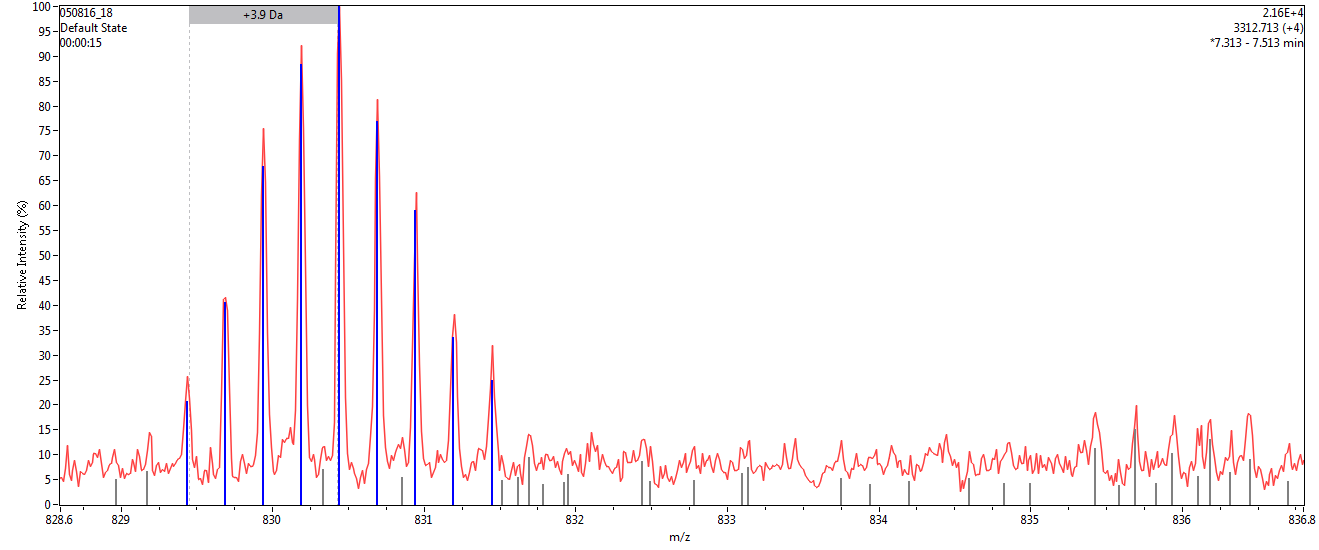


**Table S1**

| sample | 1.8 mg/ml | | 1.5 mg/ml | | 1 mg/ml | | 0.75 mg/ml | |
| --- | --- | --- | --- | --- | --- | --- | --- | --- |
| Instrument | BioXolver L with GeniX3D X-ray source | | | | | | | |
| Wavelength [Å] | 1.54 | | | | | | | |
| Sample volume [µl] | 7 | | 7 | | 7 | | 7 | |
| sample-detector distance [mm] | 571 | 1382 | 571 | 1382 | 571 | 1382 | 571 | 1382 |
| number of frames | 30 | 40 | 60 | 40/30 | 60 | 60 | 90 | 60 |
| exposure time per frame [s] | 60 | 60 | 60 | 60/120 | 60 | 120 | 60 | 120 |
| total measurement time [min] | 30 | 40 | 60 | 100 | 60 | 120 | 90 | 120 |

**Table S2**

| SOFTWARE | **data reduction** | RAW^3,4^ |
| --- | --- | --- |
|  | **IFT** | GNOM^5^ |
|  | ***ab initio* modeling** | DAMMIF, DAMFILT^6^ |
|  | **3D representation** | PyMOL |
| Guinier | **number of data points used for Guinier approximation** | 10 |
|  | **q-range used for Guinier approximation [Å^-1^]** | 0.0056 – 0.0102 |
|  | **q_max_ × R_g_** | 0.90 |
|  | **radius of gyration R_g_ (ΔR_g_) [Å]** | 88.41 (33.42) |
|  | **I(0)/c (ΔI(0)/c) [cm^2^/mg]** | 8.39×10^-2^ (1.52×10^-2^) |
|  | **MW (ΔMW) from absolute scale [kDa]** | 152 (28) |
| GNOM | **number of data points used for GNOM fit** | 168 |
|  | **q-range used for GNOM fit [Å^-1^]** | 0.0056 - 0.2 |
|  | **D_max_ × q_min_** | 1.51 |
|  | **number of points in P(r)** | 101 |
|  | **radius of gyration R_g_ (ΔR_g_) [Å]** | 85.34 (1.65) |
|  | **longest extension D_max_ (ΔD_max_) [Å]** | 296 |
|  | **I(0)/c (ΔI(0)/c) [cm^2^/mg]** | 6.69×10^-2^ (1.69×10^-3^) |
|  | **Χ^2^** | 2.1 |
|  | **MW (ΔMW) from absolute scale [kDa]** | 121 (3) |
| DAMMIF | **symmetry/anisometry** | P1/prolate |
|  | **mode** | slow |
|  | **number of reconstructions** | 5 |
|  | **NSD *** | 1.322 ± 0.060 |

* The NSD value describes the similarity between the generated models. A value above ∼1 indicates that the models are dissimilar.

**Table S3**

| Surface |  | *k*_a_ x 10^6^  (1/Ms) | *k*_d_  (1/s) | K_D_  (pM) | R_max_  (RU) |
| --- | --- | --- | --- | --- | --- |
| KDM5B (881 RU) |  | 1.068±0.002 | 8.73±0.05e-4 | 817±5 | 60.92±0.02 |

**References**

1. Wriggers, W. Conventions and workflows for using *Situs*. *Acta Crystallogr. Sect. D Biol. Crystallogr.* **68,** 344–351 (2012).

2. Ortega, A., Amorós, D. & García de la Torre, J. Prediction of Hydrodynamic and Other Solution Properties of Rigid Proteins from Atomic- and Residue-Level Models. *Biophys. J.* **101,** 892–898 (2011).

3. Hopkins, J. B., Gillilan, R. E. & Skou, S. BioXTAS RAW: Improvements to a free open-source program for small-angle X-ray scattering data reduction and analysis. *J. Appl. Crystallogr.* **50,** 1545–1553 (2017).

4. Nielsen, S. S. *et al.* BioXTAS RAW , a software program for high-throughput automated small-angle X-ray scattering data reduction and preliminary analysis. *J. Appl. Crystallogr.* **42,** 959–964 (2009).

5. Svergun, D. I. Determination of the regularization parameter in indirect-transform methods using perceptual criteria. *J. Appl. Crystallogr.* **25,** 495–503 (1992).

6. Franke, D. *et al.* *ATSAS 2.8* : a comprehensive data analysis suite for small-angle scattering from macromolecular solutions. *J. Appl. Crystallogr.* **50,** 1212–1225 (2017).
